# Improving executive function in childhood: evaluation of a training intervention for 5-year-old children

**DOI:** 10.3389/fpsyg.2015.00525

**Published:** 2015-04-30

**Authors:** Laura Traverso, Paola Viterbori, Maria Carmen Usai

**Affiliations:** Department of Education Sciences, University of GenoaGenoa, Italy

**Keywords:** executive function, training, preschool, inhibition (psychology), working memory, cognitive flexibility

## Abstract

Executive function (EF) refers to a set of higher order cognitive processes that control and modulate cognition under continuously changing and multiple task demands. EF plays a central role in early childhood, is associated and predictive of important cognitive achievements and has been recognized as a significant aspect of school readiness. This study examines the efficacy of a group based intervention for 5-year-old children that focuses on basic components of EF (working memory, inhibitory control, cognitive flexibility). The intervention included 12 sessions, lasted 1 month and used low-cost materials. Seventy-five children took part in the study. The results indicate that the children who attended the intervention outperformed controls in simple and more complex EF tasks. Specifically, these children exhibited increased abilities to delay gratification, to control on-going responses, to process and update information, and to manage high cognitive conflict. These results suggest the possibility that this intervention, which may be easily implemented in educational services, can promote EF during preschool period before the entrance in primary school.

## Introduction

Executive function (EF) refers to a set of cognitive abilities that allow individuals to control thoughts and actions in the face of new or complex situations in which an automatic or impulsive response is not useful (see Miyake and Friedman, 2012). These functions help individuals select the most advantageous choice when confronted with the complex and heterogeneous demands of life and include skills such as the ability to suppress inappropriate responses (inhibition), the ability to flexibly shift between ideas and activities (cognitive flexibility), and the capacity to hold, to update and actively manipulate information in mind (working memory) (Miyake et al., 2000). In addition to this traditional cognitive model, an emotional component of EF has also been conceptualized. Zelazo and Müller (2002) have made a distinction between the development of relatively “hot” emotional aspects of EF and the development of more purely “cool” cognitive aspects. Whereas, cool EF is likely to be elicited by relatively abstract and context-free problems, hot EF is required in situations that involve the regulation of affect and motivation. Cool EF is evoked in situations or activities that are cognitively demanding and emotionally neutral (e.g., retrieving information after being manipulated mentally, such as during a working memory task); hot EF is elicited in situations where there is motivational involvement, such as when a reward is expected. It has been suggested that hot and cool EF typically work together as part of a more general adaptive system (Zelazo and Carlson, 2012).

## The role of EF during development

Although EF develops over a long period of time that spans from the first year of life until late adolescence, the most impressive change in EF skills occurs during the preschool period (Garon et al., 2008). The rapid growth in EF that takes place between the ages of 3 and 5 enables children to organize their thinking and behavior with increasing flexibility, decrease their reactive responding to contextual cues, and engage in self-regulated and rule-governed behavior (for a review see Garon et al., 2008).

Individual variations in the development of EF within this age range have been found to be associated with and predictive of important cognitive achievements, such as self regulation (Sokol and Müller, 2007), social competence, specifically the Theory of Mind (Hughes and Ensor, 2007), and learning abilities (Blair and Razza, 2007). EF deficits have been found in several psychopathological conditions such as Attention-Deficit Hyperactivity Disorder (ADHD, Castellanos et al., 2006), pervasive developmental disorders (Pellicano, 2012), intellectual disabilities (Lanfranchi et al., 2009), and learning difficulties (Andersson and Lyxell, 2007).

In particular, EF development is significantly related to a child's learning ability (Bull et al., 2008; Brock et al., 2009). The relationship between early EF and later school achievements is fairly robust. Longitudinal research has suggested that EF skills contribute significantly to both mathematical and literacy achievement (Bull and Scerif, 2001; Blair and Razza, 2007; Clark et al., 2010) in children of various ages with and without specific learning disabilities (Müller et al., 2008; Best et al., 2009). As reported by Wass (2015) early individual differences in cognitive control capacity may mediate the later-emerging differences in learning skills and academic outcomes in typical development (Snyder and Munakata, 2011) and in atypical conditions, such as children from low-SES backgrounds that are more likely than their peers to have reduced EF (Welsh et al., 2010), children at risk of ADHD (Lawson and Ruff, 2004) or children with genetic disorders (Cornish et al., 2012).

## Efficacy of EF intervention in children

In recent years, several types of training aimed at enhancing EF have been proposed (Diamond and Lee, 2011). Although there are still several open questions regarding the efficacy of EF interventions (Morrison and Chein, 2011; Shipstead et al., 2012; Melby-Lervag and Hulme, 2013), they still may represent an opportunity for children at risk for specific disorders and for clinical populations. For example, although parent training and medical treatment are the most common clinical approaches to ADHD (Klingberg et al., 2005; Charach et al., 2013) tested a working memory computer training program for ADHD children (RoboMemo, Cogmed Cognitive Medical Systems AB, Stockholm, Sweden) that showed positive results for working memory and transfer effects on inhibition performance that were maintained at follow up.

Though the promising results of EF interventions (Klingberg et al., 2005; Holmes et al., 2009; Diamond and Lee, 2011), most studies were focused on school age children (8–12 years, Diamond and Lee, 2011), sometimes with contradictory results (no EF gains in children with autistic spectrum disorder, Fisher and Happe, 2005; a positive effect in children with intellectual disabilities, Söderqvist et al., 2012).

Thus far, to our knowledge, only a limited number of studies have investigated the effect of EF intervention on preschool children (e.g., 4–6 years), despite the potential preventive effect of early intervention (Sonuga-Barke and Halperin, 2011). Actually, as suggested by Melby-Lervag and Hulme (2013) younger children may show significantly larger benefits from training than older children and the promotion of EF development during the preschool period could increase the school readiness of children (Blair, 2002).

Regarding preschool interventions, different types of program have been developed for typical children. Comparing the effects of these interventions using a cost-benefit approach is difficult because they differ in duration (long- vs. short-term interventions), setting (individual vs. group interventions), and materials.

The long-term programs are generally group-based interventions that correspond to a school curriculum and are provided in educational services over the entire length of the preschool or during the year before the primary school entrance (e.g., Bierman et al., 2008a,b; Raver et al., 2011). An example of such an intervention is Tools of the Mind curriculum developed by Bodrova and Leong (1996) based on a Vygotskian approach. The program emphasizes the development of underlying skills such as paying attention, remembering on purpose, logic, and symbolic representation; opportunities to learn cognitive and socio-emotional self-regulation abilities are interwoven into almost all classroom activities throughout the day. In a randomized trial, Diamond et al. (2007) found that preschoolers from low-income families who attended the Tools of the Mind Program showed markedly better EF performance than control group.

Nevertheless, the implementation of this type of intervention have some strict requirements, such as time resource, the commitment of school principals, intensive teacher training and good student–teacher ratios (see also Lillard and Else-Quest, 2006; Domitrovich et al., 2007). The need for such resources can make these programs expensive and can reduce their feasibility.

Short-term interventions are generally individualized training to be carried out over periods ranging from 1 week to 1 month (see Appendix A for a review). They include several computer-based trainings with rather intensive time schedules lasting from 2 to 5 weeks with 2 to 5 sessions per week (Rueda et al., 2005, 2012; Thorell et al., 2009; Bergman Nutley et al., 2011) or paper-and-pencil activities with three to eight short sessions concentrated in a week (Dowsett and Livesey, 2000; Kloo and Perner, 2003). Finally, a mixed individual and group training that use different types of activities and games has been proposed by Röthlisberger et al. (2011). Their intervention focused on the basic components of EF—i.e., working memory, interference control and cognitive flexibility—and represents a good trade-off between individualized computer-based interventions and large-group curriculum interventions. Prekindergarten (5-year-old) and kindergarten children (6-year-old) were involved in daily sessions of approximately 30 min in which three different tasks were performed in three different ways: in a group, in pairs of children and individually. The tasks were adapted versions of some well-known EF tasks (e.g., Simon says, Luria's hand game, dimensional card sorting, listening recall). Activities were carried out twice a week by a trained experimenter and by the teachers, who were trained and supervised by the experimenter, on the remaining 3 days.

Although short-term interventions differ in terms of training procedures and the EF components targeted, they have generally proven to be effective in promoting working memory (Bergman Nutley et al., 2011; Röthlisberger et al., 2011, for only prekindergarten children; Thorell et al., 2009) and cognitive flexibility (Kloo and Perner, 2003; Röthlisberger et al., 2011 only for prekindergarten children). Regarding interference control, the results are rather mixed. One study found significant training effects in preschool children with poor inhibitory skills (Dowsett and Livesey, 2000), another study found this effect only in kindergarten children (Röthlisberger et al., 2011), and three studies of typically developing children failed to find any increase (Rueda et al., 2005, 2012; Thorell et al., 2009). Finally, to our knowledge, only the study by Rueda et al. (2012) found a partially positive effect of the training on hot EF, which was still present at follow-up.

The results of studies on early EF interventions are promising and suggest that different strategies may be useful for enhancing EF during preschool period. However, most studies documented the effectiveness of interventions only on a limited set of EFs: some studies focused on specific EFs components (such as working memory); and showed some limitation for a use in preschool educational settings.

Early EF intervention that could be implemented in educational services for preschoolers could represent a prevention strategy for children with a potential delay or impairment in the development of EF, such as children from low socioeconomic backgrounds (Noble et al., 2005; Farah et al., 2006; Kishiyama et al., 2009) or children at risk for ADHD symptoms (Diamond and Lee, 2011). This type of program could be very useful in responding to the needs of diverse populations of children that are not always adequately identified and managed during the preschool years.

Nevertheless, previous EF training programs for preschool children, though partially effective, can be challenging and expensive when applied in standard educational contexts. Most programs are highly resource-consuming because, in some cases, they require the specific training of teachers and in the other cases the interventions are based on short-term individualized activities that should be conducted under the supervision of a trained adult.

## The present study

The aim of the present study was to evaluate the efficacy of a training program designed to promote EF during preschool period, in particular in 5-year-old children that are present in educational services before to start the primary school at age of six, by following a cost-effective approach suitable for educational services. The key point is whether a play-based group training that can be easily implemented in school settings, including low-resource contexts, may be as effective as other, more expensive types of interventions, to increase EF. To develop a training, suitable for educational services that requires low cost material and low time and personnel resources, may be a strategy to reduce the gap in EF level through children at risk, such as children from disadvantage social condition, before school entrance.

No computers or other technical equipment was used, and all required materials were simple, inexpensive, and readily available. The training activities were completely separate from the assessment tasks to avoid the observation of any apparent increases in EF in the training group that may have resulted from intensive practice on the assessment tasks rather than real improvements in EF.

To demonstrate the efficacy of the training in promoting EF, an extensive battery was used to assess the three core EF components (i.e., inhibition, shifting, and working memory). The tasks were selected to evaluate growing levels of cognitive control and both the hot and cool aspects of EF. We expected the intervention to be effective in improving different EF abilities, such as inhibition, working memory and flexibility, immediately after the end of the training.

## Methods

### Participants

Five-year-old children who attended four public educational services (kindergarten) in commonly recognized disadvantage areas in the main province of a northwestern region of Italy were enrolled for this study. In Italy, children from 3 to 5 years old attend kindergarten that offer a pre-primary curriculum, that emphasizes activities that enhance creativity, social attitudes, autonomy, and learning, and it supports school readiness. Kindergartens are mostly public and free of charge for families, except for lunch fees, which depend on family income. Attendance at kindergartens is non-compulsory, but it is almost universal: more than 95% of the target children attend kindergarten before to start the primary school at age of six. Depending on the school, classes are age-homogeneous or age-heterogeneous; in the latter case, 3 to 5 year old children share most of the educational activities, except for lab activities, in which only one age group is included at a time. Additionally, in the case of age-homogeneous classes, small-group activities are equally common. Classes are composed maximum of 29 children. For this study, the priority was given to schools serving areas commonly recognized as low-income ones in which most of the children attended full time.

**Figure 1:**
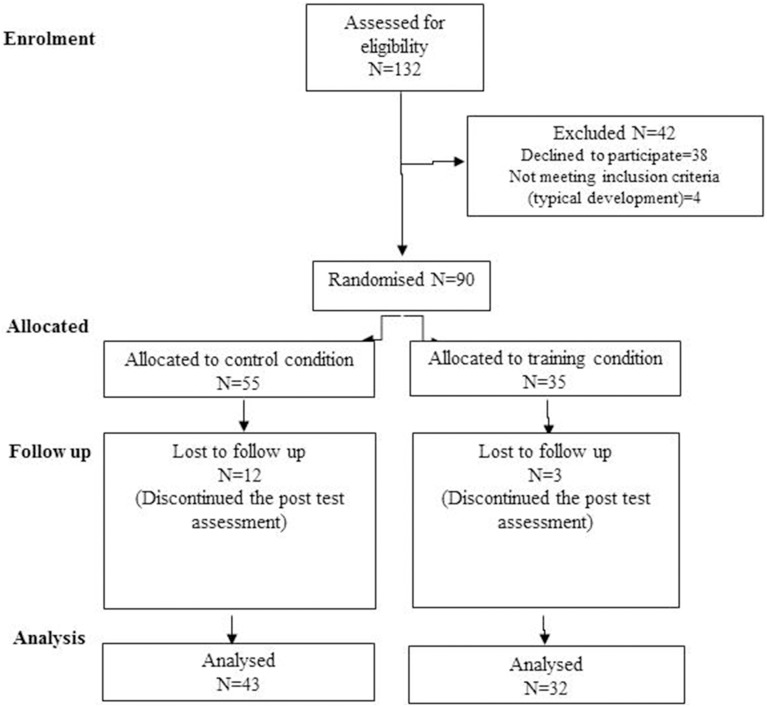
**Participants flow chart**.

As shown in Figure 1, the project was presented to 132 families; 38 parents refused to give consent to participation, and 4 children were excluded due to ascertained developmental disorders Children with special needs or disabilities are fully integrated into the regular classroom, nevertheless we preferred to initially verify the efficacy of our training in children with typical development. The parents of the remaining 90 children filled up the parental informed consent and provided information about their socio-demographic conditions and their children's behavior by completing two brief questionnaires. The 90 children were assigned randomly to the training and control groups. Five children were selected at random from each class to be included in the training group, whereas the remaining children formed the control group. This procedure was adopted to guarantee that the control and experimental children shared the same school setting and that school context could not differently affect EF development. At the end of the random assignment, the training group and the control group consisted of 35 and 55 children, respectively.

In contrast to other studies (Rueda et al., 2005; Thorell et al., 2009), we did not include both active and passive control groups for the following two reasons: no differences emerged from the comparisons of the performances of passive and active groups in previous studies (Rueda et al., 2005; Thorell et al., 2009), and we wanted to compare this training with standard kindergarten activities, which usually include small-group activities for children of the same age.

At the post-intervention assessment, 15 children (12 controls and 3 experimental children) did not complete the evaluation due to prolonged absence from school and were consequently excluded from the analyses. The 15 children who were excluded from the study did not differ from the others with regard to their socio-demographic characteristics. All of the children included in the intervention attended at least 8 out of 12 sessions.

The final sample consequently consisted of 75 children ranging in age from 62 to 76 months (*M*_age_ = 68.6; SD = 3.5; 53% female): 43 children comprised the control group (*M* = 68.6; SD = 3.6; age range: 62–75 month; 58% female), and 32 children comprised the training group (*M* = 68.7; SD = 3.5; range: 63–76 months; 47% female).

### Procedure

Pre-test assessments were conducted for both the control and training groups. Following the pre-test, which lasted approximately 2 weeks, a month of training within the regular kindergarten day commenced for the intervention group only. After training, all of the children were immediately reassessed no later than 2 weeks following the end of the training.

All tasks described in the following section were administered twice (i.e., pre- and post-training), with the exception of the Colored Progressive Matrices (CPM, Raven, 1947). The CPM were used as a screening measure to verify that there were no difference for intelligence at baseline for the two groups. In both the pre- and post-training conditions, the children were tested individually by trained psychologists, blind to children condition, over three sessions that lasted approximately 20–25 min each. The assessments took place at school in a silent room during the kindergarten day. The tasks were administered in a fixed order for two main reasons. First, a fixed order is preferred for the investigation of individual differences (see Carlson and Moses, 2001; Wiebe et al., 2008), and fixed orders allow for the control of session duration and the variation of tasks according to the materials, response modalities and abilities required. The order in which tasks were administered, as well as a summary of the variable labels, is reported in Table 1.

**Table 1:**
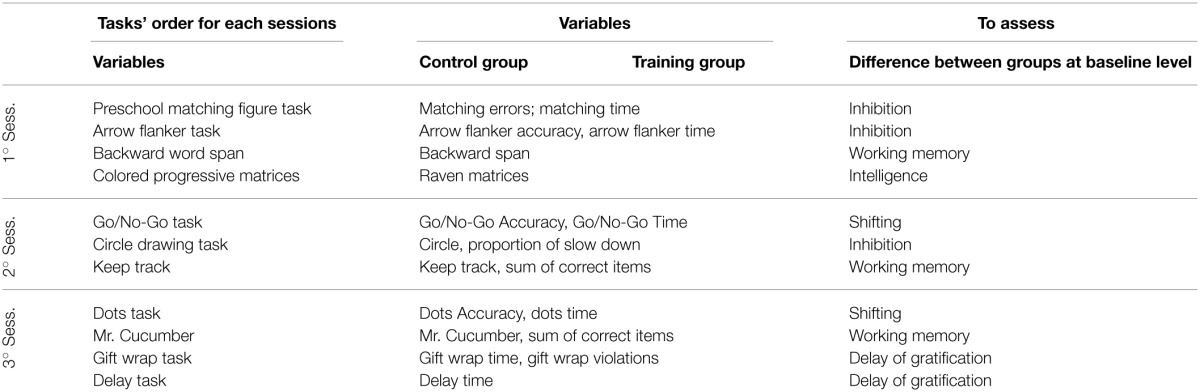
**Summary of the assessment battery: the order of tasks for each session and the variables labels used in each task to assess EF and cognitive abilities are reported**.

### Measures

#### EF assessment

Tasks were selected based on the following criteria: 1. All tasks required the children to actively control their reactions. Impulsive or automatic responses led to mistakes. 2. Most of the tasks were well-known EF measures in child research. 3. The tasks were chosen to minimize the effects of non-executive abilities, such as the children's vocabulary and knowledge, and the instructions were simple, involved familiar materials and required different types of input and output modalities (i.e., verbal/visuospatial stimuli, hot/cool situations, motor/verbal responses, and pencil paper/computer tasks). 4. All tasks differed from the training activities. 5. The tasks required different levels of control the ranged from simple motor control to conditions of high cognitive conflict. Most of the tasks had multiple codings, such as time and accuracy.

#### Hot EF tasks

To assess hot aspects of EF, two delay tasks were used.

#### Delay task

This task (adapted from Kochanska et al., 1996) is a version of the standard delay paradigm that is frequently used to assess the ability of children to delay gratification (see Kochanska et al., 2000). The child is asked to wait as long as she can before opening a gift box and the latency is recorded (Delay Task Time, expected range 0-no limit).

#### Gift wrap task

This task (Kochanska et al., 1996) is used to evaluate the ability to delay gratification and inhibit undesirable behaviors in children (Carlson and Moses, 2001; Carlson, 2005). Children were told that the examiner would wrap a present behind their back and that they should not peek until the examiner said they were allowed to do so. The examiner then noisily wraps the gift over a period of 60 s. The latency to the first peek (Gift Wrap Task Time, expected range 0–60 s) and the total number of peeks during the 1-min interval were coded (Gift Wrap Violations, expected range 0-no limit).

#### Inhibition tasks

A set of different tasks was used to assess inhibition.

#### Circle drawing task

This task (Bachorowski and Newman, 1985) is a well-known measure of the motor inhibition of an on-going response that has been used for both adult (Wallace et al., 1991) and childhood assessments (Geurts et al., 2005; Marzocchi et al., 2008; Usai et al., 2014). The child must trace with his finger over a 17 cm diameter circle from a starting point to an ending point. The task is administered twice. On the first administration, neutral instructions (“trace the circle”) were given, and on the second administration inhibition instructions were given (“trace the circle again but this time as slowly as you can”). Larger time differences indicate better inhibition (slowing down) on the part of the participant in her continuous tracing response. Time in seconds was recorded for each trial. Scores were calculated as the slowdown relative to the total time using the following formula: T2−T1/T2+T1, where T1 and T2 were the times recorded for the first and second trials, respectively (Circle drawing task, expected range negative to positive values-no limit).

#### Preschool matching familiar figure task

This task (adapted by Kagan, 1966) measures the child's ability to restrain impulsive responses and to compare the target with all of the pictures by shifting attention from the target to each alternative. The child is asked to select from among different alternatives the figure that is identical to the target picture at the top of the page. In the form that has been adapted for kindergartners, this task involves five alternatives and is comprised of 14 items. The number of errors (Matching Errors, expected range 0–56) and the mean latency between the presentation of the item and the child's response were recorded (Matching Time, expected range 0-no limit).

#### Arrow flanker task

The Flanker task (adapted from Ridderinkhof and van der Molen, 1995) is a well-known paradigm that is used to evaluate the ability to inhibit irrelevant interfering stimuli (Eriksen and Eriksen, 1974; Kramer et al., 1994). The child is required to respond to a left or right pointing arrow presented at the center of the computer screen by pressing a left or right response button. The arrow is flanked by two arrows pointing in the same direction (congruent condition, 16 items) or in the opposite direction (incongruent condition, 16 items) or by two simple lines in the neutral condition (16 items). After a brief training consisting of six items (two of each condition), 48 items are randomly presented (16 items per condition, half left and half right). A warning cross (500 ms in duration) preceded the stimulus (1500 ms in duration). After the stimulus, the screen turned blank for 500 ms. Response times for each item (Arrow Flanker Time, expected range 0–3 s) and accuracies in the incongruent condition were recorded (Arrow Flanker Accuracy, expected range 0–16).

#### Shifting tasks

Two different tasks were used to assess shifting.

#### Go/No-Go task

The go/no go task (adapted from Berlin and Bohlin, 2002) is a well-known paradigm that tests the abilities of both adults and children to inhibit prepotent responses (Durston et al., 2002; Verbruggen and Logan, 2008). In the third condition, the children are asked not only to restrain an automatic response but also to pay attention to, and shift between, different dimensions of the same object. While in front of a computer screen, the child is instructed to press the space bar according to the instructions given by the examiner for the following three conditions: 1. “Press the space bar when you see a blue figure; do not press when you see a red figure.” (30 items: 12 blue stars, 12 blue balls, 3 red stars, 3 red balls); 2. “Press the space bar when you see the star; do not press when you see the ball” (30 items: 12 red stars, 12 blue stars, 3 red balls, 3 blue balls); and 3. “Press the space bar when you see a blue star, do not press for the remaining figures” (40 items, 32 blue stars, 4 blue balls, 2 red stars, 2 red balls). The percentage of go responses was 80% in each of the three conditions. The stimulus duration was 3000 ms, and the blank page that appears after each stimulus lasted 1000 ms. The sum of the correct responses in the no go conditions (Go/No-Go Accuracy, expected range 0–8) and the mean response time for all of the three conditions were calculated (Go/No-Go Time, expected range 0–3 s).

#### Dots task

This task (Diamond et al., 2007) is a high cognitive conflict task in which the child has to shift between rules according to the stimulus presented (see Diamond et al., 2007; Diamond and Lee, 2011). A heart or a flower appears on the right or left of a computer screen. The child is told that he must press on the same side as the heart but on the side opposite the flower, which requires inhibiting the tendency to respond on the side where the stimulus appeared. After a brief training session with heart and flower items, the test began, and hearts and flowers were intermixed in the test. The sum of the correct responses (Dots Accuracy, expected range 0–3 s) and the mean latency for correct responses were recorded for each child (Dots Time, expected range 0–20).

#### Working memory and updating tasks

Three tasks were used to assess children working memory and updating ability.

#### Backward word span

This task (Ciccarelli, 1998) is a traditional working memory task (Carlson, 2005; Alloway et al., 2006). This task requires the child to recall a sequence of spoken words in reverse order. Words were presented approximately once per second. After an illustration trial, the test begins with three trials of two words. The number of words increments by one every three trials until three lists are recalled incorrectly. The maximum list length at which two sequences were correctly recalled was scored (Backward Span, expected range 1–9).

#### Mr. cucumber

This task (Case, 1985) is a measure of working memory in children (Morra, 1994). The examiner presents a large outline drawing of an extra-terrestrial character with a number of colored stickers attached to it at specific body parts (e.g., on the nose, on the left antler, etc.) for 5 s. The child is then shown a colorless drawing and asked to indicate the positions of the stickers in the previously presented figure. There are three items per level (from 1 to 8 stickers, in ascending order). An item is scored as correct if the child points at all of the correct body parts and no other body parts. One point is given for each consecutive level on which a child correctly indicates at least two items, and one third of a point is given for each correct item beyond that level (Mr. Cucumber, expected range 0–8).

#### Keep track

The Keep Track task (adapted by Van der Ven et al., 2011) is a working memory task that is suitable for assessing updating ability in both adults (Miyake et al., 2000) and children (Van der Sluis et al., 2007; Van der Ven et al., 2011). A computerized version of the Keep Track task was created. The child was shown pictures, each of which belonged to one of the following five categories: animals (dog, cat, fish, bird), sky (sun, moon, stars, cloud), fruit (strawberry, grapes, pear, apple), vehicles (train, bicycle, motorbike, car), and clothes (socks, skirt, t-shirt, shoes). Before each trial, the child was asked to pay special attention to one (first three trials) or two designated categories (last three trials). The pictures were shown in series of six. During the presentation of each series, the child had to name each picture. At the end, the child had to recall the last item in each designated category, which required managing the interference cause by the other named pictures. The number of designated categories increased from one (in the first three series) to two (in the last three series). During picture presentation, small pictures symbolizing the to-be-remembered categories were shown at the bottom of the screen to serve as a reminder. One point was given for each correct response, and 0.5 points were given if the child was not able to recall the item and asked to see all the pictures in the requested category again (Keep Track, expected range 0–9).

#### Fluid intelligence

The Colored Progressive Matrices Test (Raven, 1947) was administered to measure fluid intelligence and was used as a screener. It is a multiple choice test of abstract reasoning in which the child is required to complete a geometrical figure by choosing the missing piece among 6 possible drawings; the patterns progressively increase in difficulty during the 36 items presented (CPM, expected range 0–36).

#### Parent report questionnaire

Parents evaluated children using the Attention and hyperactivity symptoms scale, Parents version (Cornoldi et al., 1996) a rating scale in which parents report the prevalence of their child's inattentive behaviors (9 items) and hyperactive-impulsivity symptoms (9 items) on four-point Likert scales (from never = 0 to very frequently = 3). The scale has been validated and standardized for the Italian population and exhibits good reliability and validity (Marzocchi and Cornoldi, 2000). This scale was used in the pre-test assessment to verify that the control and training groups did not differ in their levels of dis-attention or hyperactive behavior.

### Training

The intervention program we developed aimed to foster EF skills through a series of small group game activities that require progressively higher levels of inhibitory control, working memory, and cognitive flexibility. Specifically, the intervention was proposed to small groups of five children, while the others children performed the normal kindergarten activities that include small group laboratories. The intervention was performed three times a week and it included 12 sessions of approximately 30 min each over approximately 1 month; the training took place at school in a silent room during the kindergarten day.

For the intervention, we adopted a play-based approach to intervention using the same story and characters through which the children enact roles during and across sessions to involve the children and maintain their motivation to collaborate. Specifically, in the first session, children are invited to listen to the fantasy story of Chicco and Nanà, two little goblin friends attending kindergarten. Unfortunately, the two friends have difficulty thinking carefully before acting such that, while preparing a magic potion, they erroneously transform themselves into a mouse and a cat, a condition in which it is difficult to be friends. To be converted into goblins again so that they may attend primary school, their teacher wants them to overcome 10 different challenges that will help them become more regulated. The children are asked to help Chicco and Nanà, by overcoming different challenges (intervention activities) that require EF skills.

All of the training activities were different from the assessment tasks that were administered to the children before and after the intervention, which required increasing levels of cognitive control and active participation on the part of each child. All of the activities were specifically designed for 5-year-old children so that they were challenged and engaged but also experienced a manageable level of difficulty. Each activity required that the entire group reach the fixed goals; thus, the children had to collaborate and positively reinforce each other to reach the goal (for a brief description of the training activities, see the Training Description in the Appendix B).

In order to help children manage the activities, all of the training sessions were structured in the same way so that the children could focus on the new activity without being distracted by the setting. Each activity started with a brief warm-up activity to introduce the session; then, the children were given an explanation for the new activity and were assigned their roles and tasks; finally, the session ended with a metacognitive activity.

The adult introduced the activities and the rules that all children had to respect, facilitated the interaction among the children, provided suggestions and support only when strictly necessary, and helped children to be autonomous in managing and controlling the game. Each child was given a different role with a specific responsibility—for example, the director was in charge of managing the players' behavior. During the session, the roles were exchanged. The children were invited to resolve conflicts by complying with the rules of the activity and respecting the roles they were assigned. Moreover, we provided concrete aids to help the children develop and practice self-regulation strategies through concrete experiences with physical materials. Every training session ended with a metacognitive activity that consisted of asking children to color smiling faces reported on a schedule according to their self -perception of their EF and to share strategies that they considered useful in performing the challenges. Special attention was paid to support the children's self-esteem and well-being during the activities, and the children were praised for their efforts during and at the end of each session.

The training involved low-cost, easily available materials (e.g., colored markers, pens and pencils, cardboard, paper, printed materials). The activities were designed to be included in the standard kindergarten curriculum, which emphasizes learning through play. Finally, the small-group approach, which is typically part of the standard organization in Italian kindergartens, can be easily implemented in the daily school schedule.

The training was carried out by a trained psychologist. The fidelity of training implementation was ensured by requiring to the training psychologist to know the aims of the training, how to perform the activities and to manage the situation by consulting the training book that we developed. At this level of evaluation, we decided to do not involve teachers before determining whether our training was effective. At the end of the project, teachers received a brief course in which the research findings were presented, the training books were shared and the teachers were supported in learning training aims and activities. An online version of the training book was developed to permit free download.

## Results

Descriptive analyses were conducted on pre-test and post-test data to verify the variables' distributions and the rate of missing data and outliers. Then, chi-square and *t*-test were performed to verify the existence of differences between the training and control groups at baseline on socio-demographic variables, children's symptoms of inattention and hyperactivity, and pre-test task performance (i.e., on the EF tasks and Colored Progressive Matrices). Subsequently, pre-to-post differences between the groups, using pre-test scores as covariates, were performed to investigate training efficacy. To verify the relative magnitudes of the experimental treatment, effect sizes (range: 0–1) were calculated using Cohen's (1988) effect size formula.

### Descriptive statistics for the EF tasks, considering pre- and post-tests, and reliability

Descriptive statistics (i.e., means, standard deviations, possible score ranges, skewness, and kurtosis) for the EF tasks were conducted with respect to data from the pre- and post-EF task assessments. Large interindividual variabilities were recorded for most tasks. No floor or ceiling effects were found with the exception of the first two conditions of the Go/No-Go Task. The percentage of missing values ranged from 0 to 3%, with a single exception of 9% in the third condition of the Go/No-Go Task, which was mostly attributable to the duration of the task.

Scores that deviated from the mean by 3 standard deviations (SDs) or more were considered outliers and were excluded from the analyses. Outliers comprised 0–1% of the data across all of the tasks with exception of the Delay Task, in which 6% of the data were considered outliers. All the tasks were normally distributed, with the exceptions of the Delay Task and the Preschool Matching Familiar Task (time), for which logarithmic transformations were used to obtain improved skewness and kurtosis parameters.

Pearson correlations between the control group's performance on EF tasks at the pre and post test showed that, across all tasks, the retest reliability was moderate, with a mean *r* = 0.58 (range = 0.41–0.99).

### Verifying differences between training and control groups at the baseline level before the training

No significant differences between the control and training groups were found at baseline in terms of mothers' and fathers' levels of education, parents' perceptions of social and economic support, family income, levels of inattention and hyperactive behavior as reported in the parent report questionnaires, percentage of bilingual children, presence of brothers or sisters, children's mean age, gender distribution, or general cognitive abilities (no children scored below the 25th percentile), as reported in Table 2.

**Table 2:** **Training vs. control group comparison: socio-demographic characteristics and parental reports of the children's behavior at baseline, before beginning the training**.

	**Training group**	**Control group**		
*Mother years of education*	12.53	12.31	*t*_(1, 56)_ = 0.236, *p* = 0.814	No difference
Primary school, 5 years of ed.	0	4%		
Secondary sc., 1°grade, 8 years of ed.	31%	23%		
High School Diploma, 13 years of ed.	47%	58%		
University, 18 years of ed.	22%	15%		
Master, 22 years of ed.	0	0		
*Father years of education*	12.61	10.68	*t*_(1, 54)_ = 1.947, *p* = 0.057	No difference
Primary school, 5 years of ed.	0	4%		
Secondary sc., 1°grade, 8 years of ed.	29%	48%		
High school diploma, 13 years of ed.	55%	40%		
University, 18 years of ed.	10%	8%		
Master, 22 years of ed.	7%	0%		
*Family Earn by year*			*X*^2^_(5, 43)_ = 8.545, *p* = 0.129	
≤10,000	14%	14%		No difference
10,000–15,000	17%	0		
15,000–20,000	10%	43%		
20,000–25,000	21%	7%		
25,000–30,000	17%	21%		
≥30,000	21%	14%		
*Perceived economical support*			*X*^2^_(3, 51)_ = 1.299, *p* = 0.729	
Insufficient	0	4%		No difference
Quite sufficient	19%	17%		
Acceptable	42%	46%		
Quite good	30%	33%		
Optimal	0	0		
*Perceived social support*			*X*^2^_(4, 53)_ = 1.158, *p* = 0.885	
Insufficient	17%	8%		No difference
Quite sufficient	7%	8%		
Acceptable	24%	21%		
Quite good	17%	21%		
Optimal	35%	42%		
*Family with more than one child*	65%	66%	*X*^2^_(1, 75)_ = 0.002, *p* = 0.963	No difference
*Parents report on child*				
dis-attentive behaviors	7.09	7.19	*t*_(1, 73)_ = −0.068, *p* = 0.946	No difference
hyperactive-impulsivity symptoms	6.86	6.72	*t*_(1, 73)_ = 0.109, *p* = 0.913	
*Birth in Italy*	100%	100%	–	No difference
*Italian as the first language spoken*	100%	100%	–	No difference
*Bilingual children*	16%	9%	*X*^2^_(1, 75)_ = 0.757, *p* = 0.384	No difference
*Children mean age*	68.60	68.69	*t*_(1, 73)_ = −0.100, *p* = 0.921	No difference
*Sex distribution, percentage of female*	58	47	*X*^2^_(1, 75)_ = 0.935, *p* = 0.333	No difference
*General cognitive ability (Raven Matrices)*	16.60	17.19	*t*_(1, 73)_ = −0.922, *p* = 0.360	No difference

As shown in Table 3, no differences between the control and training groups were found with respect to EF tasks performance at the pre-test assessment, with the sole exception of the Arrow Flanker Task; in this task, the control group outperformed the training group in terms of accuracy.

**Table 3:** **Training vs. control group comparison: EF tasks performance at baseline level, before the training**.

	**Training group**	**Control group**	**Difference at baseline level, pre test assesment**	
**DELAY OF GRATIFICATION**				
Delay time	1.08	1.27	*t*_(1, 66)_ = −1.768, *p* = 0.082	No difference
Gift wrap time	28.83	27.15	*t*_(1, 73)_ = 0.389, *p* = 0.698	No difference
Gift wrap vilations	2.76	2.34	*t*_(1, 69)_ = 0.856, *p* = 0.395	No difference
**INHIBITION**				
Circle	0.41	0.33	*t*_(1, 73)_ = 1.470, *p* = 0.146	No difference
Matching time	0.74	0.82	*t*_(1, 71)_ = −1.422, *p* = 0.160	No difference
Matching errors	12.98	13.06	*t*_(1, 73)_ = −0.065, *p* = 0.948	No difference
Arrow flanker accuracy	0 7.49	9.56	*t*_(1, 73)_ = −2.092, *p* = 0.040	Control > Training
Arrow flanker time	874.90	846.08	*t*_(1, 72)_ = −0.803, *p* = 0.425	No difference
**SHIFTING**				
Go/No-Go accuracy	5.61	5.2	*t*_(1, 66)_ = 0.776, *p* = 0.441	No difference
Go/No-Go time	745.36	715.26	*t*_(1, 66)_ = 0.585, *p* = 0.561	No difference
Dots accuracy	12.77	13.03	*t*_(1, 73)_ = −0.065, *p* = 0.948	No difference
Dots time	1177.75	1237.91	*t*_(1, 73)_ = −0.672, *p* = 0.819	No difference
**WORKING MEMORY**				
Backward span	1.98	2.03	*t*_(1, 72)_ = −0.378, *p* = 0.706	No difference
Mr: Cucumber	1.64	1.71	*t*_(1, 72)_ = −0.489, *p* = 0.626	No difference
Keep track	3.15	4	*t*_(1, 73)_ = −1.741, *p* = 0.086	No difference

### Results of the efficacy study

To test the efficacy of our training, we conducted a between-group comparison (training vs. control group) using analyses of covariance with the pre-test scores from each individual task covariates. This statistical technique, which combines regression analysis and analysis of variance, is preferable to the use of repeated measures analyses for experimental designs with pre- and post-tests (Dimitrov and Rumrill, 2003).

The analysis of the training efficacy revealed a significant effect of group on post-test performance after controlling for pre-test levels in the following tasks (Table 4): the Delay Task [*F*_(1, 64)_ = 8.61, *p* < 0.01]; Gift Wrap Time [*F*_(1, 73)_ = 8.41, *p* < 0.01]; the Circle Drawing Task [*F*_(1, 72)_ = 7.38, *p* < 0.01]; Preschool Matching Familiar Figure Task accuracy [*F*_(1, 73)_ = 5.10, *p* < 0.05]; Arrow Flanker Task time [*F*_(1, 70)_ = 4.14, *p* < 0.05]; Dots Task accuracy [*F*_(1, 71)_ = 6.04, *p* < 0.05]; Backswords Word Span [*F*_(1, 71)_ = 4.13, *p* < 0.05]; and Keep Track [*F*_(1, 73)_ = 8.03, *p* < 0.01].

**Table 4:** Comparison of the performances of the training and control groups in the EF tasks post-assessment: means, standard deviations, the results of between-group (control vs. training groups) analyses of covariance using pre-test scores as covariates and effect sizes are reported (^*^p < 0.05; ^**^p < 0.01).

		**Post-Training assessment**		**Group effect**		
	**Group**	**Mean**	***SD***	**F**	**Direction**	**Effect size**
**DELAY OF GRATIFICATION**						
Delay time	Control	0.83	0.62	8.61**	**Training > Control**	0.70
	Training	1.25	0.57			
Gift wrap time	Control	33.72	18.90	8.41**	***Control > Training***	0.65
	Training	22.08	16.62			
Gift wrap violations	Control	2.14	2.02	0.07	*No difference*	0.44
	Training	2.11	1.87			
**INHIBITION**						
Circle	Control	0.37	0.25	7.38**	**Training > Control**	0.35
	Training	0.46	0.26			
Matching time	Control	0.73	0.28	3.08	*No difference*	0.44
	Training	0.84	0.19			
Matching errors	Control	13.02	6.93	5.10*	**Training > Control**	.45
	Training	10.28	4.61			
Arrow flanker time	Control	888.57	7.96	4.14*	**Training > Control**	0.61
	Training	820.95	6.40			
Arrow flanker acc.	Control	11	3.93	0.42	*No difference*	0.28
	Training	12.09	3.77			
**SHIFTING**						
Go/No-Go time	Control	711.41	194.41	0	*No difference*	0.02
	Training	714.71	177.43			
Go/No-Go accuracy	Control	5.61	2.22	0	*No difference*	0.16
	Training	5.94	2.02			
Dots time	Control	1125.28	322.36	3.3	*No difference*	0.14
	Training	1168.77	292.99			
Dots accuracy	Control	12.44	3.82	6.04*	**Training > Control**	0.53
	Training	14.59	3.14			
**WORKING MEMORY**						
Backward span	Control	1.98	0.64	4.13*	**Training > Control**	0.43
	Training	2.22	0.42			
Mr. Cucumber	Control	1.83	0.62	1.54	*No difference*	0.27
	Training	2.01	0.66			
Keep track	Control	3.78	2.14	8.03**	**Training > Control**	0.65
	Training	5.34	2.69			

For all of these tasks, the results indicate that the children who took part in the training performed better than the children who only attended the standard preschool activities. The only exception was the Gift Wrap hot task, in which the control children increased their waiting time at the second assessment, whereas the training children did not. The training group outperformed the control group in most inhibitory control tasks and also in two of the three working memory tasks (Backswords Word Span and Keep Track) and the Dots Task, which required cognitive flexibility.

To verify the relative magnitudes of the experimental treatment, effect sizes were calculated using Cohen's (1988) effect size formula (*d*). Based on this formula, an effect size of 0.20 is considered small, an effect of 0.50 is considered medium, and an effect of 0.80 is considered large. As shown in Table 4, the effect sizes of the training ranged from medium to large for the majority of the tasks.

## Discussion

Several studies have confirmed the importance of EF in development (Bull et al., 2008).

However, early EF interventions have shown only partial results, and most were not developed for widespread use in preschool settings because they required trained personnel, time resources or technical equipment. Nevertheless, the development of an early EF intervention that can be easily implemented in educational services could be useful for enhancing school readiness and reducing the gap in EF development between typical and at risk children (such as children from disadvantaged contexts and those with poor working memory or suspected ADHD), especially when they are not yet properly identified.

The present study was conducted to examine the efficacy of an EF training program that was developed to be suitable for educational services using low cost materials and limited time and personnel resources. The training targets 5-year-old children attending the last year of preschool.

### Training effects on EF

The training produced positive results in all of the three principal EF components—i.e., inhibition, working memory and cognitive flexibility—whereas previous studies had found significant effects only in specific EF dimensions, such as working memory (Thorell et al., 2009). Only Röthlisberger et al. (2011) had found substantial training effects for both working memory and cognitive flexibility in a sample of 5-year-old children, while interference control improved only in a sample of 6-year-old children.

The dissimilarity between the training activities and the tasks adopted in the assessment lead us to believe we measured real improvements in EF capacity and were not observing a mere task-training effect. The training group performed better in both the simple and the more complex tasks. The training group exhibited increased inhibition abilities, particularly in the control of ongoing motor responses as measured by the Circle Drawing Task and in the control of impulsive reactions as measured by the Preschool Matching Familiar Figure Task. The training group required less time to find the correct response in the presence of interfering stimuli in the Flanker Task, exhibited enhanced working memory abilities in both the Backward Word Span and Keep Track Task, and exhibited better performance in the Dots Task, which measured both inhibition and working memory in a switching context. Regarding the latter task, Diamond (2002) indicated that this task requires the conjunction of two simultaneous demands: holding information in mind and inhibiting inappropriate responses, a combination that is truly difficult—particularly, if one's mental settings have to be continually switched according to task changes. These types of tasks thus require continuous cognitive control and are indicative of cognitive flexibility. An increase in these functions is therefore particularly significant in terms of the cognitive prerequisites for school readiness and academic performance because cognitive flexibility is significantly associated with both school achievement (see, for example, Bull et al., 1999) and superior approaches to learning that begin in the preschool period (Vitiello et al., 2011).

Regarding hot EF, the effects of the training were rather mixed, and the results suggest that the training did not consistently influence these EF components. In the Gift Wrap Task, the controls outperformed the training children, who exhibited reduced waiting time at the second assessment, whereas, in the Delay Task, the opposite pattern was found: the control children performed worse at the second assessment. Both of the tasks that we used to evaluate hot EF are associated with the ability to cope with frustration. Although the children were asked to manage their negative feelings somewhat—for example, while waiting their turn to provide an answer—during the training activities, this aspect was not specifically addressed by the training.

The training children outperformed the control group in the majority of the EF tasks. This study demonstrates that it is possible to enhance EF skills using an ecological training in which 5-year-old children are engaged in a series of group play based activities. The ecological setting may be particularly useful in reducing regulation difficulties due to EF deficit such as in ADHD children. This type of training indeed stimulates children to regulate themselves during and through playing with peers. The use of a real life situation such as playing in a preschool setting could be useful to help children generalize the cognitive improvements at least to other similar situations or tasks. For now, in fact, there is no convincing evidence of the generalization of WM and EF training to other skills for both typically and atypically developing children (Melby-Lervag and Hulme, 2013; Rapport et al., 2013).

### Limitations and future directions

Three major limitations of this study should be noted. First, the training was administered by a trained psychologist. To verify the effectiveness and generalizability of this training, evaluation of the training as administered by teachers is required. Second, we did not evaluate whether the gains in EF shown by the training group endured over time or whether they were associated with greater school readiness or enhanced achievement at the end of kindergarten and Grade 1. Third, we did not include an active control group; although we controlled for test-retest effects, it may be important to investigate the intervention effect considering an active control group that is matched with respect to time and effort with the training group (Brehmer et al., 2012).

Nevertheless, the results of this study are promising; these results indicate that it is possible to foster the development of different aspects of EF with relatively simple interventions. Future studies might seek to investigate the transferability of this training program and the exploration of long-term effects on EF and school achievement. Given the importance of cognitive and emotion regulation for children's school adjustment, further research should also explore what could be improved in the training program to observe more consistent effects on hot EF. Finally, it may be particularly helpful to verify the effect of this type of intervention with at risk children (e.g., children from disadvantage context) or atypical children, such as children with low EF due to social disadvantage, ADHD children, learning difficulties children.

## Conclusion

In conclusion, this study confirms the efficacy of a school-based intervention that addressed all of the EF components in 5-year-old children. Differently from most intervention studies that engage school age children, this intervention focuses on preschool children. Moreover, in contrast to previous preschool interventions, this training was developed using a low-cost approach to make it feasible for educational services. Specifically, a group-based approach was preferred because it is easier to implement within the daily schedules of preschool settings than individualized interventions. Second, we preferred the use of easily available materials to ensure that the intervention may also be suitable for educational services located in disadvantaged and low-resource contexts, in which children are at higher risk of poor EF.

Given the predictive association between EF and later achievement, interventions that begin in preschool period may lead to better outcomes, especially among children who are at risk, because they may experience increase school readiness and thereby reduce the achievement gap associated with socioeconomic disadvantage (Lawson et al., 2013; Nesbitt et al., 2013). In conclusion, the development of low-cost EF training that could be feasible for educational settings should be considered a priority for prevention research.

### Conflict of interest statement

The authors declare that the research was conducted in the absence of any commercial or financial relationships that could be construed as a potential conflict of interest.

## References

[B1] Alloway T. P. Gathercole S. E. Pickering S. J. (2006). Verbal and visuo-spatial short-term and working memory in children: are they separable? Child Dev. 77, 1698–1716. 10.1111/j.1467-8624.2006.00968.x 17107455

[B2] Andersson U. Lyxell B. (2007). Working memory deficit in children with mathematical difficulties: a general or specific deficit? J. Exp. Child Psychol. 96, 197–228. 10.1016/j.jecp.2006.10.001 17118398

[B3] Bachorowski J. A. Newman J. P. (1985). Impulsivity in adults: motor inhibition and time-interval estimation. Pers. Individ. Dif. 6, 133–136 10.1016/0191-8869(85)90041-8

[B4] Bergman Nutley S. Soderqvist S. Bryde S. Thorell L. B. Humphreys K. Klingberg T. (2011). Gains in fluid intelligence after training non-verbal reasoning in 4-year-old children: a controlled, randomized study. Dev. Sci. 14, 591–601. 10.1111/j.1467-7687.2010.01022.x 21477197

[B5] Berlin L. Bohlin G. (2002). Response inhibition, hyperactivity and conduct problems among preschool children. J. Clin. Child Adolesc. Psychol. 31, 242–251. 10.1207/S15374424JCCP3102_09 12056107

[B6] Best J. R. Miller P. H. Jones L. L. (2009). Executive functions after age 5: changes and correlates. Dev. Rev. 29, 180–200. 10.1016/j.dr.2009.05.002 20161467 PMC2792574

[B7] Bierman K. L. Domitrovich C. E. Nix R. L. Gest S. D. Welsh J. A. Greenberg M. T.. (2008a). Promoting academic and social-emotional school readiness: the head start REDI program. Child Dev. 79, 1802–1817. 10.1111/j.1467-8624.2008.01227.x 19037951 PMC3549580

[B8] Bierman K. L. Nix R. L. Greenberg M. T. Domitrovich C. E. Blair C. (2008b). Executive functions and school readiness intervention: impact, moderation, and mediation in the head start-REDI program. Dev. Psychopathol. 20, 821–843. 10.1017/S0954579408000394 18606033 PMC3205459

[B9] Blair C. (2002). School readiness: integrating cognition and emotion in a neurobiological conceptualization of child functioning at school entry. Am. Psychol. 57, 111–127. 10.1037/0003-066X.57.2.111 11899554

[B10] Blair C. Razza P. C. (2007). Relating effortful control, executive function, and false belief understanding to emerging math and literacy ability in kindergarten. Child Dev. 78, 647–663. 10.1111/j.1467-8624.2007.01019.x 17381795

[B11] Bodrova E. Leong D. J. (1996). Tools of the Mind: The Vygotskian Approach to Early Childhood Education. Englewood Cliffs, NJ: Merrill/Prentice Hall.

[B12] Brehmer Y. Rieckmann A. Bellander M. Westerberg H. Fischer H. Backman L. (2012). Neural correlates of training-related working-memory gains in old age. Neuroimage 58, 1110–1120. 10.1016/j.neuroimage.2011.06.079 21757013

[B13] Brock L. L. Rimm-Kaufman S. E. Nathanson L. (2009). The contributions of ‘hot’ and ‘cool’ executive function to children's academic achievement and learning-related behaviors, and engagement in kindergarten. Early Child. Res. Q. 24, 337–349 10.1016/j.ecresq.2009.06.001

[B14] Bull R. Espy K. A. Wiebe S. A. (2008). Short-term memory, working memory, and executive functioning in preschoolers: longitudinal predictors of mathematical achievement at age 7 years. Dev. Neuropsychol. 33, 205–228. 10.1080/87565640801982312 18473197 PMC2729141

[B15] Bull R. Johnson R. S. Roy J. A. (1999). Exploring the roles of the visuo-spatial sketchpad and central executive in children's arithmetical skills: views from cognition and developmental neuropsychology. Dev. Neuropsychol. 15, 421–442 10.1080/87565649909540759

[B16] Bull R. Scerif G. (2001). Executive functioning as a predictor of children's mathematics ability: inhibition, shifting and working memory. Dev. Neuropsychol. 19, 273–293. 10.1207/S15326942DN1903_3 11758669

[B17] Carlson S. M. (2005). Developmentally sensitive measures of executive function in preschool children. Dev. Neuropsychol. 28, 595–616. 10.1207/s15326942dn2802_3 16144429

[B18] Carlson S. M. Moses L. J. (2001). Individual differences in inhibitory control and children's theory of mind. Child Dev. 72, 1032–1053. 10.1111/1467-8624.00333 11480933

[B19] Case R. (1985). Intellectual Development from Birth to Adulthood. Orlando, FL: Academy Press.

[B20] Castellanos F. X. Sonuga-Barke E. J. S. Milham M. P. Tannock R. (2006). Characterizing cognition in ADHD: beyond executive function. Trends Cogn. Sci. 10, 117–123. 10.1016/j.tics.2006.01.011 16460990

[B21] Charach A. Carson P. Fox S. Ali M. U. Beckett J. Lim C. G. (2013). Interventions for preschool children at high risk for ADHD: a comparative effectiveness review. Pediatrics 131, 1584–1604. 10.1542/peds.2012-0974 23545375

[B22] Ciccarelli L. (1998). Language Comprehension, Processing and Working Memory: A Study with Preschoolers. Unpublished dissertation thesis, University of Padua, Italy.

[B23] Clark C. A. C. Pritchard V. E. Woodward L. J. (2010). Preschool executive functioning abilities predict early mathematics achievement. Dev. Psychol. 46, 1176–1191. 10.1037/a0019672 20822231

[B86] Cohen J. (1988). Statistical Power Analysis for the Behavioral Sciences. New York, NY: Routledge Academic.

[B24] Cornish K. Cole V. Longhi E. Karmiloff-Smith A. Scerif G. (2012). Does attention constrain developmental trajectories in Fragile X syndrome? A 3-year prospective longitudinal study. Am. J. Intellect. Dev. Disabil. 117, 103–120. 10.1352/1944-7558-117.2.103 22515826

[B25] Cornoldi C. Gardinale M. Masi A. Pettenò L. (1996). Impulsività e Autocontrollo: Interventi e Tecniche Metacognitive. Trento, Erickson.

[B87] Diamond A. (2002). Normal development of prefrontal cortex from birth to young adulthood: cognitive functions, anatomy, and biochemistry, in Principles of Frontal Lobe Function, eds Stuss D. T. Knight R. T. (New York, NY: Oxford University Press), 466–503 10.1093/acprof:oso/9780195134971.003.0029

[B27] Diamond A. Barnett W. S. Thomas J. Munro S. (2007). Preschool program improves cognitive control. Science 318, 1387–1388. 10.1126/science.1151148 18048670 PMC2174918

[B28] Diamond A. Lee K. (2011). Interventions shown to aid executive function development in children 4-12 years old. Science 333, 959–964. 10.1126/science.1204529 21852486 PMC3159917

[B29] Dimitrov D. M. Rumrill P. (2003). Pretest-posttest designs in rehabilitation research. Work 20, 159–165 Available online at: http://cehd.gmu.edu/assets/docs/faculty_publications/dimitrov/file5.pdf 12671209

[B30] Domitrovich C. E. Cortes R. Greenberg M. T. (2007). Improving young children's social and emotional competence: a randomized trial of the preschool PATHS program. J. of Prim. Prev. 28, 67–91. 10.1007/s10935-007-0081-0 17265130

[B31] Dowsett S. Livesey D. (2000). The development of inhibitory control in preschool children: effects of executive skills training. Dev. Psychobiol. 36, 161–174. 10.1002/(SICI)1098-2302(200003)36:2<161::AID-DEV7>3.0.CO;2-0 10689286

[B32] Durston S. Thomas M. T. Yang Y. Ulug A. Zimmerman R. D. Casey B. J. (2002). A neural basis for development of inhibitory control. Dev. Sci. 5, 9–16 10.1111/1467-7687.00235

[B33] Eriksen B. A. Eriksen C. W. (1974). Effects of noise letters upon the identification of a target letter in a nonsearch task. Percept. Psychophys. 16, 143–149 10.3758/BF03203267

[B34] Farah M. J. Shera D. M. Savage J. H. Betancourt L. Giannetta J. M. Brodsky N. L.. (2006). Childhood poverty: specific associations with neurocognitive development. Brain Res. 1110, 166–174. 10.1016/j.brainres.2006.06.072 16879809

[B35] Fisher N. Happe F. (2005). A training study of theory of mind and executive function in children with autistic spectrum disorders. J. Autism Dev. Disord. 35, 757–771. 10.1007/s10803-005-0022-9 16283087

[B36] Garon N. Bryson S. E. Smith I. M. (2008). Executive function in preschoolers: a review using an integrative framework. Psychol. Rev. 134, 31–60. 10.1037/0033-2909.134.1.31 18193994

[B37] Geurts H. M. Verte S. Oosterlaan J. Roeyers H. Sergeant J. (2005). ADHD subtypes: do they differ in their executive functioning profile? Arch. Clin. Neuropsychol. 20, 457–477. 10.1016/j.acn.2004.11.001 15896560

[B38] Holmes J. Gathercole S. E. Dunning D. (2009). Adaptative training leads to sustained enhancement of poor working memory in children. Dev. Sci. 12, f1–f7. 10.1111/j.1467-7687.2009.00848.x 19635074

[B39] Hughes C. Ensor R. (2007). Executive function and theory of mind: predictive relations from ages 2- to 4-years. Dev. Psychol. 43, 1447–1459. 10.1037/0012-1649.43.6.1447 18020823

[B40] Kagan J. (1966). Reflection-impulsivity: the generality and dynamics of conceptual tempo. J. Abnorm. Psychol. 71, 17–24. 10.1037/h0022886 5902550

[B41] Kishiyama M. M. Boyce W. T. Jimenez A. M. Perry L. M. Knight R. T. (2009). Socioeconomic disparities affect prefrontal function in children. J. Cogn. Neurosci. 21, 1106–1115. 10.1162/jocn.2009.21101 18752394

[B42] Klingberg T. Fernell E. Olesen P. J. Johnson M. Gustafsson P. Dahlström K.. (2005). Computerized training of working memory in children with ADHD—A randomized, controlled trial. J. Am. Acad. Child Adolesc. Psychiatry 44, 177–186. 10.1007/s10803-005-0022-9 15689731

[B43] Kloo D. Perner J. (2003). Training transfer between card sorting and false belief understanding: helping children apply conflicting descriptions. Child Dev. 74, 1823–1839. 10.1046/j.1467-8624.2003.00640.x 14669898

[B44] Kochanska G. Murray K. T. Harlan E. T. (2000). Effortful control in early childhood: continuity and change, antecedents, and implications for social development. Dev. Psychol. 36, 220–232. 10.1037/0012-1649.36.2.220 10749079

[B45] Kochanska G. Murray K. T. Jacques T. Y. Koenig A. L. Vandegeest K. A. (1996). Inhibitory control in young children and its role in emerging internalization. Child Dev. 67, 490–507. 10.2307/1131828 8625724

[B46] Kramer A. F. Humphrey D. G. Larish J. F. Logan G. D. Strayer D. L. (1994). Aging and inhibition: beyond a unitary view of inhibitory processing in attention. Psychol. Aging 9, 491–512. 10.1037/0882-7974.9.4.491 7893421

[B47] Lanfranchi S. Carretti B. Spanò G. Cornoldi C. (2009). A specific deficit in visuospatial simultaneous working memory in Down syndrome. J. Intellect. Disabil. Res. 53, 474–483. 10.1111/j.1365-2788.2009.01165.x 19396941

[B48] Lawson G. M. Duda J. T. Avants B. B. Wu J. Farah M. J. (2013). Associations between children's socioeconomic status and prefrontal cortical thickness. Dev. Sci. 16, 641–652. 10.1111/desc.12096 24033570 PMC3775298

[B49] Lawson K. R. Ruff H. A. (2004). Early focused attention predicts outcome for children born prematurely. J. Dev. Behav. Pediatr. 25, 399–406. 10.1097/00004703-200412000-00003 15613988

[B50] Lillard A. Else-Quest N. (2006). Evaluating montessori education. Science 313, 1893–1894. 10.1126/science.1132362 17008512

[B51] Marzocchi G. M. Cornoldi C. (2000). Una scala di facile uso per la rilevazione di comportamenti problematici dei bambini con deficit di attenzione e iperattività. Psicol. Clin. delle Sviluppo, 4, 43–64.

[B52] Marzocchi G. M. Oosterlaan J. Zuddas A. Cavolina P. Geurts H. Redigolo D.. (2008). Contrasting deficits on executive functions between ADHD and reading disabled children. J. Child Psychol. Psychiatry 49, 469–572. 10.1111/j.1469-7610.2007.01859.x 18400060

[B53] Melby-Lervag M. Hulme C. (2013). Is working memory training effective? A meta-analytic review. Dev. Psychol. 49, 270–291. 10.1037/a0028228 22612437

[B54] Miyake A. Friedman N. P. (2012). The nature and organization of individual differences in executive functions: four general conclusions. Curr. Dir. Psychol. Sci. 21, 8–14. 10.1177/0963721411429458 22773897 PMC3388901

[B55] Miyake A. Friedman N. P. Emerson M. J. Witzki A. H. Howerter A. Wager T. D. (2000). The unity and diversity of executive functions and their contributions to complex “Frontal Lobe' task: a latent variable analysis. Cogn. Psychol. 41, 49–100. 10.1006/cogp.1999.0734 10945922

[B56] Morra S. (1994). Issues in working memory measurement: testing for M capacity. Int. J. Behav. Dev. 17, 143–159 10.1177/016502549401700109

[B57] Morrison A. Chein J. (2011). Does working memory training work? The promise and challenges of enhancing cognition by training working memory. Psychon. Bull. Rev. 18, 46–60. 10.3758/s13423-010-0034-0 21327348

[B58] Müller U. Zelazo P. D. Lurye L. E. Liebermann D. P. (2008). The effect of labeling on preschool children's performance in the dimensional change card sort. Cogn. Dev. 23, 395–408 10.1016/j.cogdev.2008.06.001

[B59] Nesbitt K. T Baker-Ward L. Willoughby M. T. (2013). Executive function mediates socio-economic and racial differences in early academic achievement. Early Child. Res. Q. 28, 774–783 10.1016/j.ecresq.2013.07.005

[B60] Noble K. G. Norman M. F. Farah M. J. (2005). Neurocognitive correlates of socioeconomic status in kindergarten children. Dev. Sci. 8, 74–87. 10.1111/j.1467-7687.2005.00394.x 15647068

[B61] Pellicano E. (2012). The development of executive function in autism. Autism Res. Treat. 2012:146132. 10.1155/2012/146132 22934168 PMC3420556

[B62] Rapport M. D. Orban S. A. Kofler M. J. Friedman L. M. (2013). Do programs designed to train working memory, other executive functions, and attention benefit children with ADHD? A meta-analytic review of cognitive, academic, and behavioral outcomes. Clin. Psychol. Rev. 33, 1237–1252. 10.1016/j.cpr.2013.08.005 24120258

[B63] Raven J. C. (1947). Progressive Matrice, Set A, Ab, B, Board and Book Form. London: H. K. Lewis: trad. it. Progressive matrici colorate, Firenze, Organizzazioni Speciali, 1954.

[B64] Raver C. C. Jones S. M. Li-Grining C. Zhai F. Bubb K. Pressler E. (2011). CSRP's impact on low-income preschoolers' preacademic skills: self-regulation as a mediating mechanism. Child Dev. 82, 362–378. 10.1111/j.1467-8624.2010.01561.x 21291447 PMC3682645

[B65] Ridderinkhof K. R. van der Molen M. W. (1995). A psychophysiological analysis of developmental differences in the ability to resist interference. Child Dev. 66, 1040–1056 10.2307/1131797

[B66] Röthlisberger M. Neuenschwander R. Cimeli P. Michel E. Roebers C. M. (2011). Improving executive functions in 5- and 6-year-olds: evaluation of a small group intervention in prekindergarten and kindergarten children. Infant Child Dev. 21, 411–429 10.1002/icd.752

[B67] Rueda M. R. Checa P. Combita L. M. (2012). Enhanced efficiency of the executive attention network after training in preschool children: immediate and after two month effects. Dev. Cogn. Neurosci. 2, 192–204. 10.1016/j.dcn.2011.09.004 22682908 PMC6987678

[B68] Rueda M. R. Rothbart M. K. McCandliss B. D. Posner P. (2005). Training, maturation, and genetic influences on the development of executive attention. Proc. Natl. Acad. Sci. U.S.A. 102, 14931–14936. 10.1073/pnas.0506897102 16192352 PMC1253585

[B69] Shipstead Z. Redick T. S. Engle R. W. (2012). Is working memory training effective? Psychol. Bull. 138, 628–654. 10.1037/a0027473 22409508

[B70] Snyder H. R. Munakata Y. (2011). Becoming self-directed: abstract representations support endogenous flexibility in children. Cognition 116, 155–167. 10.1016/j.cognition.2010.04.007 20472227 PMC2900525

[B71] Söderqvist S. Nutley S. B. Ottersen J. Grill K. M. Klingberg T. (2012). Computerized training of non-verbal reasoning and working memory in children with intellectual disability. Front. Hum. Neurosci. 6:271. 10.3389/fnhum.2012.00271 23060775 PMC3462333

[B72] Sokol B. F. Müller U. (2007). The development of self-regulation: toward the integration of cognition and emotion. Cogn. Dev. 22, 401–405 10.1016/j.cogdev.2007.08.008

[B73] Sonuga-Barke E. J. S. Halperin J. M. (2011). Developmental phenotypes and causal pathways in attention deficit/hyperactivity disorder: potential targets for early intervention? J. Child Psychol. Psychiatry 51, 368–389. 10.1111/j.1469-7610.2009.02195.x 20015192

[B74] Thorell L. B. Lindqvist S. Bergman Nutley S. Bohlin G. Klingberg T. (2009). Training and transfer effects of executive functions in preschool children. Dev. Sci. 12, 106–113. 10.1111/j.1467-7687.2008.00745.x 19120418

[B75] Usai M. C. Viterbori P. Traverso L. De Franchis V. (2014). Latent structure of executive function in 5-to and 6-year-old children: a longitudinal study. Eur. J. Dev. Psychol. 11, 447–462 10.1080/17405629.2013.840578

[B76] Van der Sluis S. de Jong P. F. van der Leij A. (2007). Executive functioning in children, and its relations with reasoning, reading, and arithmetic. Intelligence 35, 427–449 10.1016/j.intell.2006.09.001

[B77] Van der Ven S. H. G. Kroesbergen E. H. Boom J. Leseman P. P. M. (2011). The development of executive functions and early mathematics: a dynamic relationship. Br. J. Educ. Psychol. 82, 100–119. 10.1111/j.2044-8279.2011.02035.x 22429060

[B78] Verbruggen F. Logan G. D. (2008). Automatic and controlled response inhibition: associative learning in the go/no-go and stop-signal paradigms. J. Exp. Psychol. Gen. 137, 649–672 10.1037/a0013170 18999358 PMC2597400

[B79] Vitiello V. E. Greenfield D. B. Munis P. J'Lene G. (2011). Cognitive flexibility, approaches to learning, and academic school readiness in head start preschool children. Early Educ. Dev. 22, 388–410 10.1080/10409289.2011.538366

[B80] Wallace J. F. Newman J. P. Bachorowski J. A. (1991). Failures of response modulation: impulsive behavior in anxious and impulsive individuals. J. Res. Pers. 25, 23–44. 10.1016/0092-6566(91)90003-98255955

[B81] Wass S. V. (2015). Applying cognitive training to target executive functions during early development. Child Neuropsychol. 21, 150–166. 10.1080/09297049.2014.882888 24511910 PMC4270409

[B82] Welsh J. A. Nix R. L. Blair C. Bierman K. L. Nelson K. E. (2010). The development of cognitive skills and gains in academic school readiness for children from low-income families. J. Educ. Psychol. 102, 43–53. 10.1007/s10803-005-0022-9 20411025 PMC2856933

[B83] Wiebe S. A. Espy K. A. Charak D. (2008). Using confirmatory factor analysis to understand executive control in preschool children: I. Latent structure. Dev. Psychol. 44, 575–587. 10.1037/0012-1649.44.2.575 18331145

[B84] Zelazo P. D. Carlson S. M. (2012). Hot and cool executive function in childhood and adolescence: development and plasticity. Child Dev. Perspect. 6, 354–360 10.1111/j.1750-8606.2012.00246.x

[B85] Zelazo P. D. Müller U. (2002). Executive function in typical and atypical development, in Handbook of Childhood Cognitive Development, ed Goswami U. (Oxford: Blackwell Publisher), 445–469.

