# Supplementary material for: Improving executive function in childhood: evaluation of a training intervention for 5-year-old children
Source: Front Psychol. 2015 Apr 30;6:525. doi: 10.3389/fpsyg.2015.00525 (PMC4415324; doi:10.3389/fpsyg.2015.00525)
Supplement: Supplementary file 1 [file DataSheet1.DOCX]

|  | ***Aims*** | ***Sample*** | ***Group Distribution***  Appendix A *Training effects comparison* | ***Training Program*** | ***Assessment***  ***(pre-post test)*** | ***Results:***  ***Training Effect **** | ***Conclusion*** |
| --- | --- | --- | --- | --- | --- | --- | --- |
| Dowsett & Livesey, 2000 | Exp.2:  Examine the effects on inhibitory control of two different interventions for preschoolers with poor inhibition. | *N*=49,  *Age*:  3.2-5.1 years    *Recruitment:*  9 preschools in a lower-to-middle socioeconomic region, Australia. | *Assignment:*  Random  *Groups:*  Training  Practice (training on the same go/no-go task used in the assessment)  Passive control  Each group was composed of 8-24 children. | *Duration*: 3 sessions of 15-20 minutes during three day.  *Materials*: Pencil paper materials  *Activities*:  Practice group: practice on Go-no go discrimination learning task used in the assessment  Training group: practice on a simplified version of the Wisconsin Card Sorting Task and the Stop Signal Task  *Setting and adult role:* Individual interaction. The experimenter gave positive and negative feedback. | *Inhibition:* Go-no go discrimination learning task  *Follow up: N*one | The training condition was responsible  for the greatest improvement in inhibitory control. | *Inhibition: Positive results* |
| Kloo & Perner 2003 | Exp.2: Investigate the possibility of improving DCCS performance by proposing easy card sorting tasks or Theory of Mind tasks and *vice versa.* | *N*=44,  *Age*:  3-4.7 years  *Recruitment:* Five nursery schools in Salzburg and suburbs. Children were predominantly from middle class. | *Assignment*: Random controlled for pre-test performance.  *Groups:*  Card sorting training  False belief training  Control (training on other cognitive task)  Each group was composed of 14-15 children. | *Duration*: 15 minutes, for approximately 8 days +/- 2,53.  *Materials*: Pencil paper materials  *Activities*:  Card Sorting Task training: card sorting task  False belief training: theory of mind story  Control group: familiarisation with number conservation; a modified version of relative-clause task  *Setting and adult role:*  Individual interaction.  The experimenter gave feedback based on performance and provided explanations. | *Shifting:* Card Sorting Task  *Theory of Mind:* False Belief Task  *Cognitive Task*: relative-clause task (half of the sample); Number conservation task (the other half of the sample).  *Follow up: N*one | This experiment showed that card-sorting training with feedback and explanations markedly improved  children’s performances on the DCCS task compared with a control group.  Looking at the EF training transfer effects: The card-sorting group showed a significant improvement on the false belief tasks. | *Shifting: Positive results* |
|  | ***Aims*** | ***Sample*** | ***Group Distribution*** | ***Training Program*** | ***Assessment*** | ***Results**** | ***Conclusion*** |
| Rueda *et al*., 2005 | Investigate how a specific educational training targeted at the executive network might influence its development. | Exp. 1 *N*=24,  *Age*: 4 years  Exp. 2 *N*=25,  *Age*: 4 years  Exp. 3 *N*=26,  *Age*: 6 years  *Recruitment:* From a database of births in the Springfield,  OR, area. Parents received a monetary reward | *Assignment:* Random  *Groups:*  Exp. 1  Passive control  Training  Exp. 2  Active control (watch video)  Training  Exp. 3  Active control  Training  Each group was composed of 12-13 children. | *Duration*: 5 days of training for 2-3 weeks  *Materials*: Computer  *Activities*:  3 Catch Exercises  2 Anticipation exercises  1 Stimulus discrimination exercises  1 Conflict resolution task  1 Stroop exercises  1 (Just for six years old) Inhibitory exercises  Difficulties change when children pass levels  *Setting and adult role:*  Individual Interaction*.* | *Attention/Inhibition:* Child ANT  *Intelligence:* K-Bit  *Temperament:* CBQ  *Brain Activity:* EEG  *Genotype* (six years old only)  *Follow up: N*one | No effect of training on Child Ant.  *Looking at the EF training transfer effects:* Improvement on QI task and change in EEG were observed for the four year-old group only. | *Inhibition: No effect* |
| Thorell *et al.,* 2009 | Investigate the effectiveness of two trainings: one for visuospatial WM and one for inhibition. | *N*=65,  *Age*: 4-5 years  *Recruitment:* Four different Swedish preschools (1 for an active control group; 1 for a passive control group; 2 for training groups). | *Assignment:* Random, matched for age and gender  *Groups:*  WM training  Inhibition training  Active control (computer games)  Passive control  In the analysis, the control groups were combined into a single group  Each group was composed of 14-18 children. | *Duration*: 15 minutes, every school day for 5 weeks  *Materials*: Computer  *Activities*:  WM training: 5 tasks (remember location and order of visuospatial stimuli)  Inhibitory training: 5 tasks (two go/no-go tasks; two versions of the stop signal task; flanker task)  Only three tasks were administered daily using a fixed schedule, with growing difficulty. An identical interface gave rewards and feedback for correct response.  *Setting and adult role:*  Individually interaction.  The experimenter gave positive and negative feedback. | *Inhibition:* Day-Night Stroop, Boys/Girls Stroop, Go/no-go Task  *WM:* Span Board task, Word span  *Auditory attention:* CPT  *Problem solving:* Blok Design task  *Follow up*: None | WM training group outperformed control in WM tasks. No effects were found for inhibitory training.  *Looking at the EF training transfer effects:*  No effect for the inhibition training.  The WM group exhibited only a marginal gain in go no go omissions (inhibition) and improved in CPT omission error (attention). | *Inhibition: No effect*  *Working Memory: Positive results* |
|  | ***Aims*** | ***Sample*** | ***Group Distribution*** | ***Training Program*** | ***Assessment*** | ***Results**** | ***Conclusion*** |
| Bergman Nutley *et al.,* 2011 | Investigate whether fluid intelligence is improved through non-verbal reasoning (NVR) computerised training; if NVR training or WM training result in any transfer to measure of the non-trained construct. | 1° Exp.  *N*= 112,  *Age*= 4-4.5 years  *Recruitment:* Subjects were recruited through preschools, flyers, lab webpage and advertisements in the local newspapers in Stockholm. | *Assignment:* Random (stratification by gender)  *Groups:*  WM training  NVR training  Combined training (WM+NVR)  Active Control (Combined training fixed at lowest level)  Each group was composed of 24-27 children. | *Duration*: 15 minutes at day for 5 weeks=25 sessions  *Materials: C*omputer  *Activities*:  For WM training, the same as Thorell *et al.,* 2009.  For NVR training, an algorithm generates new reasoning problems based on tasks used for assessment.  For Combined training, two WM tasks and two NVR tasks  Task difficulties change according children abilities.  *Setting and adult role:*  Individual Interaction  Parent’s support and supervision. | *WM:* Visuospatial grid task, The Odd one out,  Word Span task.  *NVR:* Repeated Patterns, Sequential Orders (Leiter)  Classification (Leiter), *CPM,* Block Design (WPPSI)  *Follow up:* None | The WM and the combined training groups improved on The odd one out.  Only WM training group outperformed Control one (although with a marginally effect) in the Word Span Task.  *Looking at the EF training transfer effects:*  WM training had no effect.  Combined training outperformed control group in Qi latent factor. | *Working Memory: Positive Results* |
| Rothlisberger *et al.,* 2011 | Examine the effects of a small group intervention developed to promote EF in children from two different kindergarten grades. | 1°Exp.(Prekindergarten) *N*=71, *Age*: 4.6-5.7 years  2° Exp. (Kindergarten) *N*=64, *Age*: 5-6.9 years  *Recruitment:* prekindergarten and kindergarten in 22 school areas with heterogeneous socioeconomic backgrounds in Swiss. | *Assignment:* Random  *Groups:*  1° Exp.(five years)  Passive control (normal school day activities)  Training  2° Exp. (six years)  Passive control (normal school day activities)  Training  Each group was composed of 30-38 children. | *Duration:* 30 daily session spread over 6 weeks  *Materials: P*encil paper kit  *Activities:* Games based on 19 traditional EF tasks (e.g., Stroop), to improve inhibition (interference control), working memory and flexibility.  *Setting and adult role:*  Individual, couple, and group setting.  The experimenter introduces for the first two days a task that teacher continues to implement in the further three days. | *Inhibition:* Simple Flanker Task  *WM:* Complex Span Task  *Flexibility:* Mixed Flanker Task  (e-prime computer based task)  *Follow up:* None | Pre-kindergarten training group outperformed the control group in the Complex Span Task and in accuracy in the Mixed Flanker Task, but no significant effect was present in the Simple Flanker Task.  At age six, the training group outperformed controls in the Simple Flanker Task but not in the Complex Span Task or Mixed Flanker Task. | *Inhibition: Partially positive effect*  *Working Memory: Partially positive effect*  *Flexibility: Partially positive effect* |
|  | ***Aims*** | ***Sample*** | ***Group Distribution*** | ***Training Program*** | ***Assessment*** | ***Results**** | ***Conclusion*** |
| Rueda *et al.,* 2012 | Examine the effect of educational training targeted at the executive network on attention and brain function.  Examine possible transfer effects to untrained abilities (regulation of affect and intelligence) | 1° Exp.  N=37,  Age: 5 years  *Recruitment:* an urban Primary School in Granada (Spain). Parents received money for participation. | *Assignment:* Pseudorandom controlled for gender, IQ, Child ANT pre test performance.  *Groups:*  Training  Active Control (watched video)  Each group was composed of 18-19 children. | *Duration=*: 45 minutes, two times a week, for 10 times  *Materials: C*omputer  *Activities: A* total of 11 exercises for Tracking/Anticipatory;  Attention Focusing/Discrimination;  Conflict Resolution;  Inhibitory Control Exercises;  Sustained Attention.  Increasing levels of difficulty.  *Setting and adult role:*  Individual Interaction. | *Attention/Inhibition:* Child ANT  *Intelligence:* K-Bit  *Temperament:* CBQ  *Hot EF:* Delay of Gratification Children Gambling task  *Brain Activity:* EEG  *Genotype* (six years old only): swabs by using the BucalAmp DNA extraction kit  *Follow up:* Two months later | No effect on Child ANT  Training Group significantly improved in Gambling not in Delay.  *Looking at the EF training transfer effects:*  Improvement in QI at post-test (matrices only) and at the follow up.  Effect on brain activation at post test and follow up | *Inhibition: No effect*  *Hot EF: Partially positive effect* |

* Note: Only the results concerning the effectiveness of EF training programs are reported: Improvements in performing EF tasks that differ from training activities (effect on non trained task) and effects of EF training on tasks assessing other abilities, such as intelligence (transfer effect). Anova and then T-test for comparison or Ancova (with pre test performance as covariate) were the principal analysis used in these studies.

Appendix B

*The training description*

**Chicco and Nanà adventures: 12 activities to promote executive function in kindergartners**

*Activity 1. Chicco, Nanà and The Magic Song*

*Reinforced Skills*: Children’s EF awareness

*Game description:* Children are invited to listen to a fantasy story that will link all of the activities in which two little goblins need children’s help to overcome ten challenges that will enhance their ability to use EFs. Children have to decide if they want to help the goblins; then, they have to learn a magic song with words and acts that will support them in remembering to use EFs and overcoming the challenges.

All of the following activities start and end in the same way. The adult asks children why they are there and what they will have to do; then, the challenge is presented. During the challenge, each child receives a role (i.e., director, referee, player) that will change over the course of the game, and everybody knows that the group must win the game (i.e., individual success are not sufficient). After the game activity, the adult asks the children to perform a metacognitive activity in which the children evaluate their effort and discuss useful strategies for performing new and complex situations.

*Activity 2. Chicco, Nanà and the Magic Frog*

*Reinforced Skills:* All EFs, mostly inhibition

*Game description:* Children must help the Magic Frog become better able to inhibit irrelevant information and control its actions. The director has to regulate attention in naming a series of pictures on a paper, and he requires to the players to touch the floor or jump according to what they hear and what they have as assigned pictures. The referee must assign a score only if all the players move correctly.

Activity 3. Chicco, Nanà and the Magic Potion

*Reinforced Skills:* All EFs, mostly inhibition and working memory

*Game description:* Children must help the goblins in making potions—i.e., be able to inhibit irrelevant ingredients, control actions and remember instructions. The director shows the players a paper in which several objects of several colors are presented. Players must remember only their color’s ingredients while all walk slowly together along a straight line, stopping if they here a bell ringing, finding the objects in a box and finally carrying them at the same time to the referee.

*Activity 4. Chicco, Nana and the Forgetful Elephant*

*Reinforced Skills*: All EFs, mostly inhibition and working memory

*Game description:* The children must help a Forgetful Elephant find its way back home and be able to remember it in the future. The children have to listen to the Elephant story, remember it and then find the correct story figures that represent the route the elephant must take to return home. Then, each child presents his or her own journey, and the players must individualize the figures that correspond to the way in which the child returns home.

*Activity 5. Chicco, Nanà and the Magic Mirror*

*Reinforced Skills:* All EF, mostly inhibition, working memory and shifting

*Game description:* The children must win the Magic Mirror to proceed in helping the goblins. The Magic Mirror does not reflect images or movements; it says what it wants to see and how. The child with the Mirror shows movements to the players that they have to remember and replicate according to the Magic Mirror’s rules.

Activity 6. Chicco, Nanà and the Magician’s House

*Reinforced Skills:* All EFs, mostly inhibition, working memory and shifting

*Game description:* To help goblins children need to go in a magician’s house. The door will open only if they are able to color the image of the house exactly as the magician says. At first, children must win pencils and papers performing a game in which they have to stop or jump according to different rules when they listen and see some color. If all children perform correctly the game, they receive pencils and papers to color the house painting remembering what the magician says.

Activity 7 Chicco, Nanà and the Magician

*Reinforced Skills:* All EFs, mostly inhibition, working memory and shifting

*Game description:* To find the Magician, the children have to be able to remember colors and forms of characteristics. The children are invited to play a memory game in which one has the picture of a secret character and the others have to discover which player has the same. Children have to look at their characters’ pictures and then cover them while remembering their features and colors.

Activity 8, Chicco, Nanà and the Magician’s first challenge

*Reinforced Skills:* All EFs, mostly inhibition and controlling actions

*Game description:* The Magician will help the goblins only if he is sure that they have become better able to use EFs. To confirm this, he asks the children to perform four challenges: the first one is to show to the Magician that the children have great inhibitory control in filling up a magic bottle. Each child, when he or she assumes the player role, has to perform different games proposed by the other child that are positioned in different places.

Activity 9, Chicco, Nanà and the Magician’s second challenge

*Reinforced Skills:* All EFs, mostly inhibition, controlling the stimuli relevance

*Game description:* Second, the children have to show to the Magician that they have great attentional control—i.e., that they are able to focus on what is relevant and inhibit other stimuli and flexibly change their mental set when what was irrelevant become relevant and vice versa. Visual and auditory stimuli are proposed, and each child that has to follow different rules. Each child will help the others to correct their performance and supporting each other’s.

Activity 10, Chicco, Nanà and the Magician’s third challenge

*Reinforced Skills:* All EFs, mostly working memory

*Game description:* The Magician wants to know if the children have good memories. A special memory card game is proposed in which children have to follow magic rules that require inhibition, shifting ability and, most of all, memory capacity that requires updating and managing information in their minds.

Activity 11, Chicco, Nanà and the Magician’s fourth challenge

*Reinforced Skills:* All EFs, mostly shifting and planning

*Game description:* The last challenge requires that children demonstrate the ability to think before acting. First, each child has to win some pieces of a puzzle by performing a game that requires inhibition, working memory and shifting, proposed by the others children, and then, they have to all make the puzzle together by supporting each other over a brief time period.

Activity 12, Chicco, Nanà and the goblins’ transformation!

*Reinforced Skills:* All EFs

Ga*me description:* The children have successfully overcome the ten challenges to help the two goblins. During this activity, they will discover the real feature of the goblins that, thanks to the child, will again turn them into children.

The children perform the final game, in which they have to identify which are the real goblins features, which is followed by the celebration of children.

All activities are available in the Italian language at www.autoregolazione.org.
